# A critical analysis of the combined usage of protein localization prediction methods: Increasing the number of independent data sets can reduce the accuracy of predicted mitochondrial localization

**DOI:** 10.1016/j.mito.2010.12.016

**Published:** 2011-05

**Authors:** Kieren T. Lythgow, Gavin Hudson, Peter Andras, Patrick F. Chinnery

**Affiliations:** a Institute of Human Genetics, Newcastle University, Central Parkway, Newcastle upon Tyne, NE1 3BZ, UK; b School of Computing Science, Newcastle University, Newcastle upon Tyne, NE1 7RU, UK

**Keywords:** Mitochondria, Proteome, Bioinformatics, Oxidative phosphorylation, Mitochondrial disease

## Abstract

In the absence of a comprehensive experimentally derived mitochondrial proteome, several bioinformatic approaches have been developed to aid the identification of novel mitochondrial disease genes within mapped nuclear genetic loci. Often, many classifiers are combined to increase the sensitivity and specificity of the predictions.

Here we show that the greatest sensitivity and specificity are obtained by using a combination of seven carefully selected classifiers. We also show that increasing the number of independent prediction methods can paradoxically decrease the accuracy of predicting mitochondrial localization. This approach will help to accelerate the identification of new mitochondrial disease genes by providing a principled way for the selection for combination of appropriate prediction methods of mitochondrial localization of proteins.

## 1 Introduction

Mitochondrial disorders are a major cause of human disease affecting at least 1 in 5000 of the population (Schaefer et al., 2008; Skladal et al., 2003). The clinical presentation of mitochondrial disorders is highly variable but often leads to a progressive, incurable multi-system disease causing substantial morbidity and often resulting in premature death (DiMauro and Schon, 2003). Oxidative phosphorylation (OXPHOS) is carried out by the mitochondrial respiratory chain, which is composed of over 100 polypeptide subunits arranged in five enzyme complexes on the inner mitochondrial membrane. Thirteen of the proteins involved OXPHOS are synthesized within the mitochondrion from mitochondrial DNA (mtDNA), and mtDNA mutations are a major cause of OXPHOS disease especially in adult life (Schaefer et al., 2008). However, the vast majority of OXPHOS subunits are synthesized from nuclear gene transcripts, along with a large number of proteins involved in mitochondrial biogenesis, mtDNA maintenance and expression, and respiratory chain assembly (Spinazzola and Zeviani, 2007).

Over ~ 50% of OXPHOS disorders are present in childhood with sporadic or autosomal recessive disease. Although the underlying gene defect can be indentified in many cases, the precise molecular cause remains unknown in the majority. Defining the underlying disease gene in unexplained OXPHOS disease has major implications, enabling accurate genetic counselling and prenatal diagnosis, and often revealing totally new mechanisms of disease (Bourdon et al., 2007; Bykhovskaya et al., 2004; Coenen et al., 2004; Mandel et al., 2001; Saada et al., 2003). Standard approaches include homozygosity mapping (Elpeleg et al., 2005; Hoefs et al., 2008; Spinazzola et al., 2006) and systematic re-sequencing (Mandel et al., 2001; Saada et al., 2003), or microcell mediated chromosomal transfer (Zhu et al., 1998) aimed at correcting a cellular biochemical defect. Although potentially very powerful, the identification of new disease genes remains hugely time consuming and very expensive, even with current sequencing platforms. This places constraints on the number of families that can be comprehensively investigated, and there is a need to develop new techniques which streamline the approach.

Several bioinformatic methods have been developed to identify putative mitochondrial proteins within a candidate interval, including N-terminal targeting sequence prediction, sequence homology, and phylogenetic relationships with known mitochondrial proteins. These tools have been combined with experimentally derived data sets including gene co-expression profiles and species homology using machine learning (Kumar et al., 2006; Prokisch et al., 2006), Bayesian (Calvo et al., 2006) and Hidden Markov (Kumar et al., 2006) techniques. The general approach is to identify parameters in the combined dataset that distinguish mitochondrial and non-mitochondrial proteins in an experimentally derived dataset, and to apply these algorithms to proteins of unknown function. Using this approach, the mitochondrial proteome is estimated at ~ 1000 proteins (Pagliarini et al., 2008), corresponding to ~ 1% of nuclear gene transcripts.

From first principles, the aim of the bioinformatic approach is to increase the specificity of predicting that a given protein is likely to be mitochondrial (correctly identifying true non-mitochondrial proteins), without unduly compromising the sensitivity (correctly identifying true mitochondrial proteins). This has been achieved by combining several bioinformatic prediction tools with an experimentally derived data set of known mitochondrial proteins (Calvo et al., 2006; Pagliarini et al., 2008). This is based on the assumption that the incorporation of additional independent data sets (i.e. independent predictions of mitochondrial localization of proteins) will inevitably improve the accuracy, including parameters derived from experimentally-based data sets. However, the finite size of the mitochondrial proteome places theoretical constraints on this strategy. As a general rule of thumb, a reliable prediction algorithm can only be derived using a set of known mitochondrial and non-mitochondrial proteins if the number of parameters is at least ~ 100 fold less than the size of the data set (Baum and Haussler, 1989; Hastie et al., 2009). Thus, predictions based on the analysis of > 5 independent prediction methods are likely to be unreliable (each method considered as a parameter of the combined method), and if carried out, the accuracy of the predictions is likely to vary considerably each time the work is carried out, questioning the reliability of published prediction tools (Hastie et al., 2009).

## 2 Methods

The performance of the different combinations of bioinformatic prediction tools was evaluated using 6352 known non-mitochondrial proteins and 467 known mitochondrial proteins extracted from SwissProt (www.ebi.ac.uk/swissprot/) with clear experimental evidence of sub-cellular localization (Available at http://www.staff.ncl.ac.uk/peter.andras/supplinfo_lythgowetal/). All of the prediction tools were evaluated using the same test proteins. The proteins were matched to the parameters derived from the eleven prediction tools listed in Table 1 (Calvo et al., 2006), with the addition of MitoProt and SubLoc (Claros and Vincens, 1996; Hua and Sun, 2001).

When combined, these resources address the issue of mitochondrial localization from several different angles, including: N-terminal targeting sequence (TargetP) (Bannai et al., 2002); protein domains (Pfam) (Guda et al., 2004); Cis-regulatory motifs in human/mouse orthologues (Calvo et al., 2006); *Saccharomyces cerevisiae* homology (Calvo et al., 2006); *Rickettsia prowazekii* ancestry (Calvo et al., 2006); coexpression with known mitochondrial genes in human/mouse tissue atlases (Calvo et al., 2006); mouse mitochondria detected in brain, heart, liver and kidney; transcriptional induction during mitochondrial biogenesis (Calvo et al., 2006); and MITOPRED (Hua and Sun, 2001); which is a genome-scale method for prediction of nucleus-encoded mitochondrial proteins. Although this is not a comprehensive list of the available prediction tools, these are amongst those most commonly used, and are generally accepted to perform at a high level. Using a larger group of prediction tools would compromise the approach, given the limited size of the test set of known mitochondrial and non-mitochondrial proteins.

Bioinformatic analysis was carried out using a workflow in the Taverna workbench (Hull et al., 2006; Oinn et al., 2006a), enabling automated large scale in-silico experiments using distributed services on the world-wide-web (Fig. S1) and incorporating a support vector machine (SVM, svm_light v6.02, http://svmlight.joachims.org/). The SVM approach has the advantage of incorporating an unbiased, objective weighting of the most discriminant component variables. The parameters for the SVM learning algorithm were optimized to yield the highest accuracy of prediction with the lowest number of support vectors. An accuracy of 95.76% was achieved using this algorithm the following parameters: -c 10 -b 0 -t 2 -g 0.1 -e 1.0; where c is the margin of tolerance to error, with increasing values increasing tolerance to error; and g is the width of the Gaussian associated with support vectors, with decreasing values decreasing the range of detection of similarity with support vectors.

Standard statistical methods were used to determine the mean, standard deviation, sensitivity and specificity, based on the Gaussian distribution. The false discovery rate (FDR) is the proportion of all the false predictions generated during the analysis FDR = FP / (FP + TP). FP = false positives. TP = true positives. A difference in the proportion of mitochondrial and non-mitochondrial proteins in training set when compared to the actual proportion in the human genome can bias the FDR. This can be accounted for by scaling the FDR based on the estimated number of mitochondrial proteins encoded by the nuclear genome (1500 of 21,000 genes). Using this approach, the corrected FDR, cFDR = (1-specificity) / (1 − specificity + sensitivity × [1500/21,000]), where specificity = TN / (TN + FP), SN = TP / (TP + FN), and TN = true negatives, FN = false negatives.

## 3 Results

### 3.1 Increasing the number of prediction tools can reduce the accuracy of predicted mitochondrial localization

**Fig. 1:**
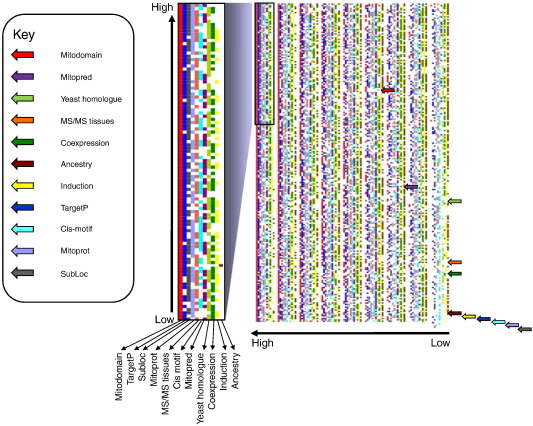
Sensitivity of different combinations of prediction tools in rank order. Left hand = detail of the top 100 combinations.

We systematically studied all possible combinations (n = 2047) of the eleven different prediction tools, using ~ 90% of the full data set as training data and ~ 10% as testing data. From an overall dataset of 6819 candidate proteins (467 mitochondrial and 6352 non-mitochondrial extracted from the Swiss-Prot database using specific localization filters, the complete list is available at http://www.staff.ncl.ac.uk/peter.andras/supplinfo_lythgowetal/), 730 were extracted from the database at random to form the test set (100 mitochondrial and 630 non-mitochondrial), leaving 6089 candidates for training. A support vector machine (SVM) was trained and tested 100 times for each one of the 2047 possible combinations of the eleven prediction parameters. Each run used a different randomly selected training and test set, allowing calculation of the mean and standard deviation of sensitivity and specificity values. The prediction results were obtained after minimizing the number of support vectors to < 10% of the training data set. Different combinations of the prediction tools were ranked based upon the mean specificity and independently for the mean sensitivity, for 100 independent SVM training runs using different randomly selected training and test sets (Fig. 1).

**Fig. 2:**
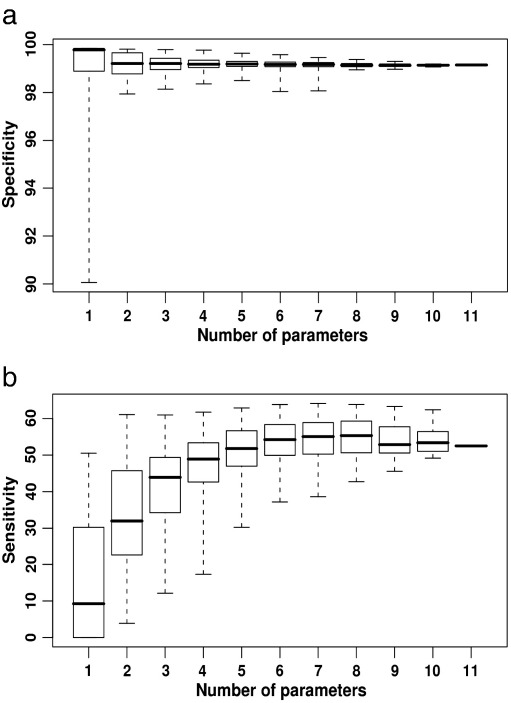
Range of mean (a) specificity and (b) sensitivity values for different combinations (n = 2047) of the eleven different prediction tools. Horizontal bar = mean, box = standard deviation, whiskers = range.

The mean specificity of the SVM runs was high (> 98%), but there was considerable variability between individual prediction tools and also between different combinations of prediction tools when combinations of < 4 were chosen (Fig. 2a). A small number of prediction tools also led to considerable variability in the sensitivity of predictions (Fig. 2b). Increasing the number of prediction tools reduced this variability, and increased the mean sensitivity without compromising the specificity (Fig. 2a).

However, increasing the number of tools beyond seven led to a decrease in mean sensitivity. Similar results were obtained for the derived false discovery rate (FDR), and corrected false discovery rate (cFDR) which incorporates prior probabilities in the calculations (Calvo et al., 2006). These findings show that increasing the number of prediction methods does not always lead to a more reliable combined prediction of mitochondrial localization.

### 3.2 Identifying the best prediction tools

**Fig. 3:**
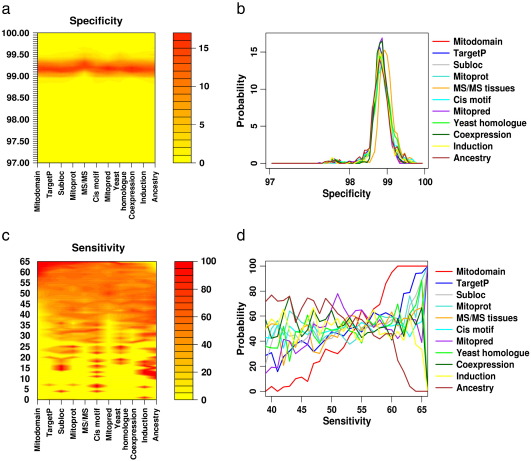
The contribution of each prediction tool to mean specificity (a and b); and mean sensitivity (c and d) for 100 training and test runs of the SVM. In this figure, “probability” refers to the proportion of combinations where a specific prediction tool contributed to a result with a given specificity and sensitivity. (a) Heat contour plot showing the probability of a specific tool or data set in contributing to a given level of specificity. Red = high probability, yellow = low probability. (b) Probability of each prediction tool contributing to a given level of specificity for the top 100 combinations. (c) Heat contour plot showing the probability of a specific tool in contributing to a given level of sensitivity. Red = high probability, yellow = low probability. (d) Probability of each prediction tool contributing to a given level of sensitivity for the prediction tools generating the top 100 mean sensitivity values.

To establish which tools improved the specificity and sensitivity of a prediction, we determined the probability of any one tool being involved in a prediction with a given specificity and sensitivity (Fig. 3; see figure legend for a detailed explanation). Although each prediction tool was equally likely to lead to a high specificity (Fig. 3a and b), this was not the case for sensitivity, where specific prediction tools were far more likely to be involved in predictions with a high sensitivity (Fig. 3c). MITODOMAIN, TargetP and MITOPRED were consistently associated with high sensitivity predictions, whilst the *R. prowazekii* orthology (ancestry) was associated with a lower sensitivity (Fig. 3d).

Careful scrutiny of the whole result set ranked from highest to lowest sensitivity (Fig. 1) confirmed these findings. The poor performance of the *R. prowazekii* orthology is probably due to two factors — first, the limited number of orthologous proteins found in humans and *R. prowazekii*, and second, the poor overall homology between mitochondria and *R. prowazekii* which are distantly related but share a common ancestor. The high performance of combinations including MITODOMAIN, TargetP and MITOPRED reflects the complementarity of the three bioinformatic approaches, even though two are based on Pfam domains within the protein secondary structure and one on the N-terminal mitochondrial targeting sequences. Combination with four other prediction tools was required to achieve optimal sensitivity and specificity.

### 3.3 Predicting the mitochondrial proteome

**Fig. 4:**
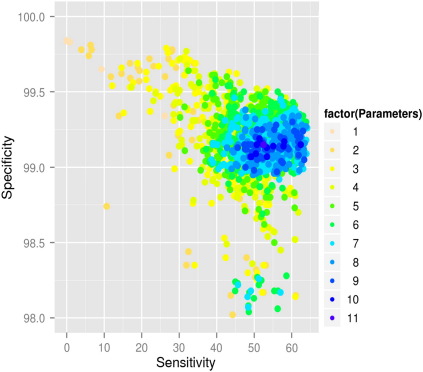
Relationship between sensitivity and specificity for the 2047 different combinations of the prediction tools. Color code = the number of prediction tools incorporated in the SVM prediction. Note the disproportionate axes.

To determine the most effective combination of prediction methods we compared the sensitivity and specificity for all 2047 combinations in (Fig. 4). Data points closest to the top right hand corner, corresponding to the best overall prediction of mitochondrial localization, were made with a combination of seven or eight tools. Incorporating a greater number of prediction tools led to a reduction in both sensitivity and specificity, confirming our earlier findings. The highest sensitivity was obtained when the following four parameters were not included the algorithm, including two based on experimentally derived data sets: yeast homology, difference in gene expression during mitochondrial biogenesis induced by PGC-1α, *R. prowazekii* orthology, and co-expression with known mitochondrial genes in human/mouse tissue atlases. The mean sensitivity using the best combination of seven prediction tools (64.14, SD = 5 .22) was significantly greater than the mean sensitivity using all 11 prediction tools (52.51, SD = 5.80). Using this tool we carried out a comprehensive genome-wide analysis of all available human transcripts (n = 37,435) from 22,258 genes available the Ensembl Genome browser to determine the predicted human mitochondrial proteome (Available at http://www.staff.ncl.ac.uk/peter.andras/supplinfo_lythgowetal/). 709 transcripts from 603 genes were predicted to localize to the mitochondrial compartment.

## 4 Discussion

The poor performance of *R. prowazekii* orthologues in isolation is well known (Calvo et al., 2006), but the work presented here shows that the incorporation of *R. prowazekii* data is actually detrimental to the process of human mitochondrial protein localization (Fig. 3d). This is probably because of the poor sequence conservation between evolutionary diverse species, which include yeast. Although much more is known about the yeast mitochondrial proteome (Andreoli et al., 2004), our observations show that this has limited relevance for the identification of putative human mitochondrial proteins, many of which are likely to have evolved after the evolutionary divergence. From first principles, the integration of data sets from lower organisms in an integrated bioinformatic prediction will thus inevitably restrict the sensitivity of detection. Our analysis shows that the inclusion of these data sets does not, however, increase specificity — providing a strong argument for excluding them from an integrated prediction approach. We were surprised that co-expression datasets, including those induced by PGC-1α, also did not substantially improve the combined prediction. This could be due to the limited number of genes incorporated in genome-wide array studies, which again would limit sensitivity without increasing specificity.

The same pattern was observed following the addition of further experimentally derived predictions of mitochondrial localization. For example, adding a log-likelihood prediction of mitochondrial localization based on subtractive MS/MS following mitochondrial enrichment (17) to the 11 prediction methods (Table 1), produced a combined mean prediction sensitivity of 50.89 (SD = 4.54) and combined mean specificity of 98.89 (SD = 0.44). This was not significantly different from the mean predictions without the subtractive MS/MS enrichment. However, removing the four prediction tools shown by our previous analysis to compromise sensitivity (Fig. 3) improved the prediction (mean sensitivity = 69.87, SD = 4.40; mean specificity = 99.23, SD = 0.34). This prediction was not significantly different from the best 7 predictors without the subtractive MS/MS enrichment, but it further demonstrates the importance of selecting the optimum combination of prediction tools based on systematic analysis.

Even with the best available combination of prediction tools, there was considerable variability in the sensitivity of the prediction each time the machine learning tool was used (Fig. S2), a feature not shown in previous reports. Although there was a general trend towards a decreased SD with combinations of more prediction tools, and higher sensitivity tended to be associated with a lower variability (Fig. S3), this relationship was not strong and the majority of predictions had an SD of ~ 5%. This means that, even for the best combination, the 95% confidence intervals for the sensitivity of predicted mitochondrial localization were ±10% of the mean value, highlighting the drawbacks of even the most optimal bioinformatic predictions. This is probably why the size of the mitochondrial proteome predicted by our approach is smaller than previously published estimates.

Our approach differs from previous attempts to bioinformatically define the mitochondrial proteome in several respects. First, SVM performance was explicitly optimized to reduce the number of support vectors to an acceptable level (Hastie et al., 2009). If the number of support vectors is greater than ~ 10% of the number of proteins in the test set, then tests of SVM performance give an impression of artificially high performance which is due to the parameters being closely tailored to the training set (overfitting). This drastically reduces the predicting capability for unknown proteins. Second, our conclusions are based on a rigorous statistically-based comparison of each prediction tool. Considerable variation between each prediction run raises the possibility that previously published combinations of predictors reported to have high sensitivity and specificity values may be spurious results. Such combinations may perform poorly over several repeated runs with independently sampled data sets. Thirdly, by developing a web-based workflow to operate the SVM, our tool will constantly update the predictions as the component prediction methods themselves improve, and the number of known human proteins increases. Thus, the list of mitochondrial proteins will be constantly revised, including revisions to the human genome sequence. This also provides the capacity to incorporate genomic variation in the predictions with increasing depth of genome re-sequencing. These combined features will accelerate the reliable identification of human disease genes responsible for novel mitochondrial disorders, providing a molecular diagnosis for families, and revealing new disease mechanisms.

### 4.1 Conclusion

The identification of a comprehensive mitochondrial proteome would be extremely advantageous both to the molecular diagnosis of mitochondrial diseases and to furthering our understanding of the complex interplay between these two genomes. In recent years several bioinformatic approaches have been developed to aid the identification of novel mitochondrial disease genes, many of which combine a number of independent prediction tools.

In this study we have demonstrated that careful selection of mitochondrial classifiers yields increased sensitivity and specificity but, perhaps more importantly, increasing the number of independent prediction methods can paradoxically decrease the accuracy of predicting mitochondrial localization.

### 4.2 Availability

Both the programs test data sets, and genome-wide predictions of mitochondrial localization are freely available at http://www.staff.ncl.ac.uk/peter.andras/supplinfo_lythgowetal/.

## Figures and Tables

**Table 1:** 

Identifier	Method	Prediction tool/data set
1	Protein domain	Pfam domain found only in eukaryotic mitochondrial proteins
2	Cis motif	Errα motif in human/mouse promoters
3	Yeast homology	*S. cerevisiae* mitochondrial orthology
4	MS/MS	Mouse mitochondria (brain, heart, liver, and kidney)
5	Induction	Difference in PGC-1α induced gene expression
6	MITOPRED	Pfam domain
7	Targeting signal	TargetP on human/mouse orthologues
8	Ancestry	*R. prowazekii* orthology
9	Coexpression	Coexpression with known human/mouse mitochondrial genes
10	Targeting signal	MitoProt II
11	Amino acid composition	SubLoc

## Appendix A Supplementary data

Fig. S1. Taverna workflow (Hull et al. 2006; Oinn et al. 2006b) used to test the different combinations of prediction tools on the training and test sets of the known mitochondrial proteins. Fig. S2. Variability in the accuracy of predictions. Standard deviation (SD, from 100 training and test runs of the support vector machine) of the sensitivity (left) and specificity (right) for the 2047 possible combinations of prediction tools. The number of prediction tools used to determine each mean prediction is color coded. Fig. S3. Relationship between the mean sensitivity and variability (shown as SD, calculated from 100 independent training and test runs of the SVM) for different combinations of seven prediction tools.
