# Supplementary figures and images for: A critical analysis of the combined usage of protein localization prediction methods: Increasing the number of independent data sets can reduce the accuracy of predicted mitochondrial localization
Source: Mitochondrion. 2011 May;11(3-2):444–9. doi: 10.1016/j.mito.2010.12.016 (PMC3081538; doi:10.1016/j.mito.2010.12.016)

Figure S1

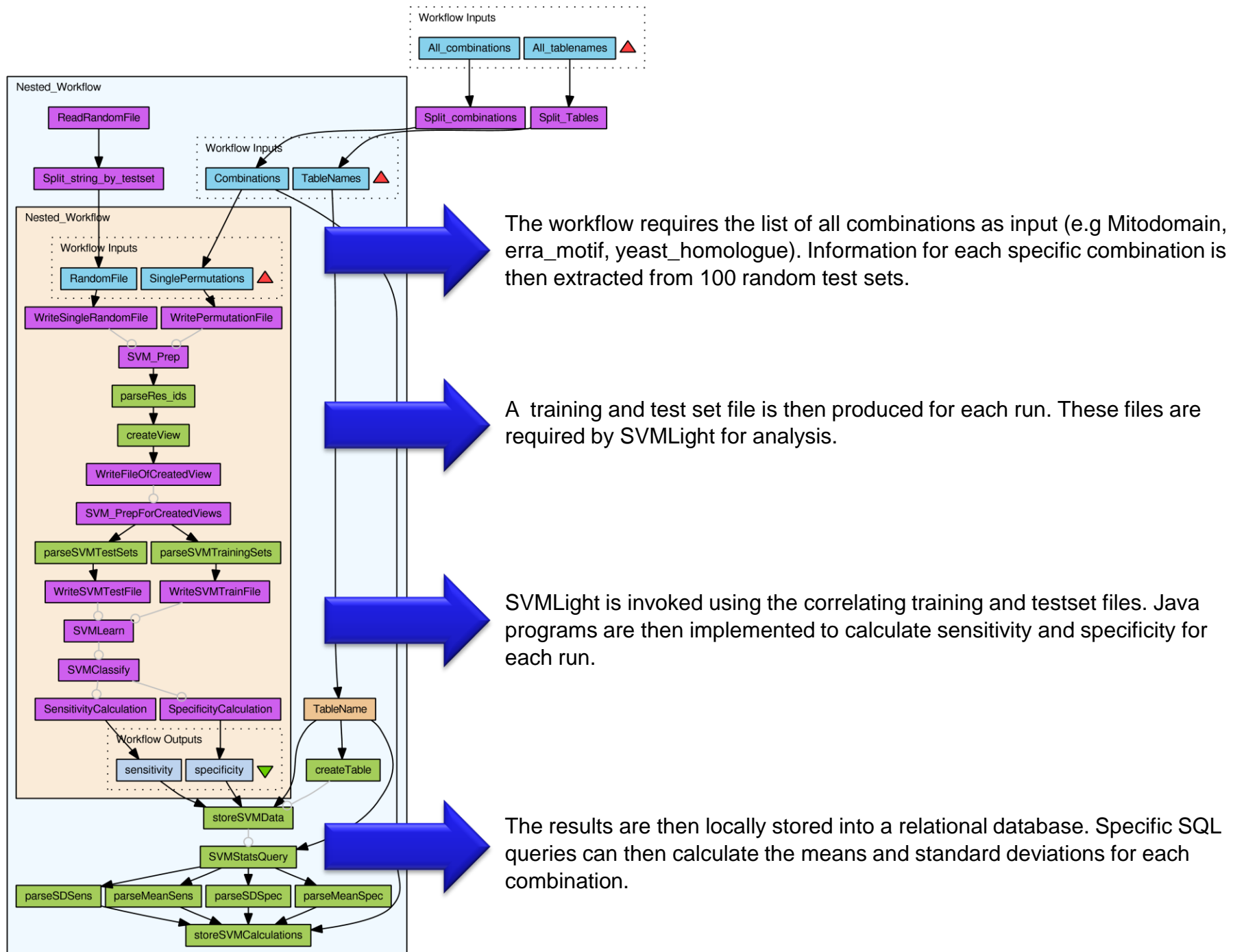

Figure S2

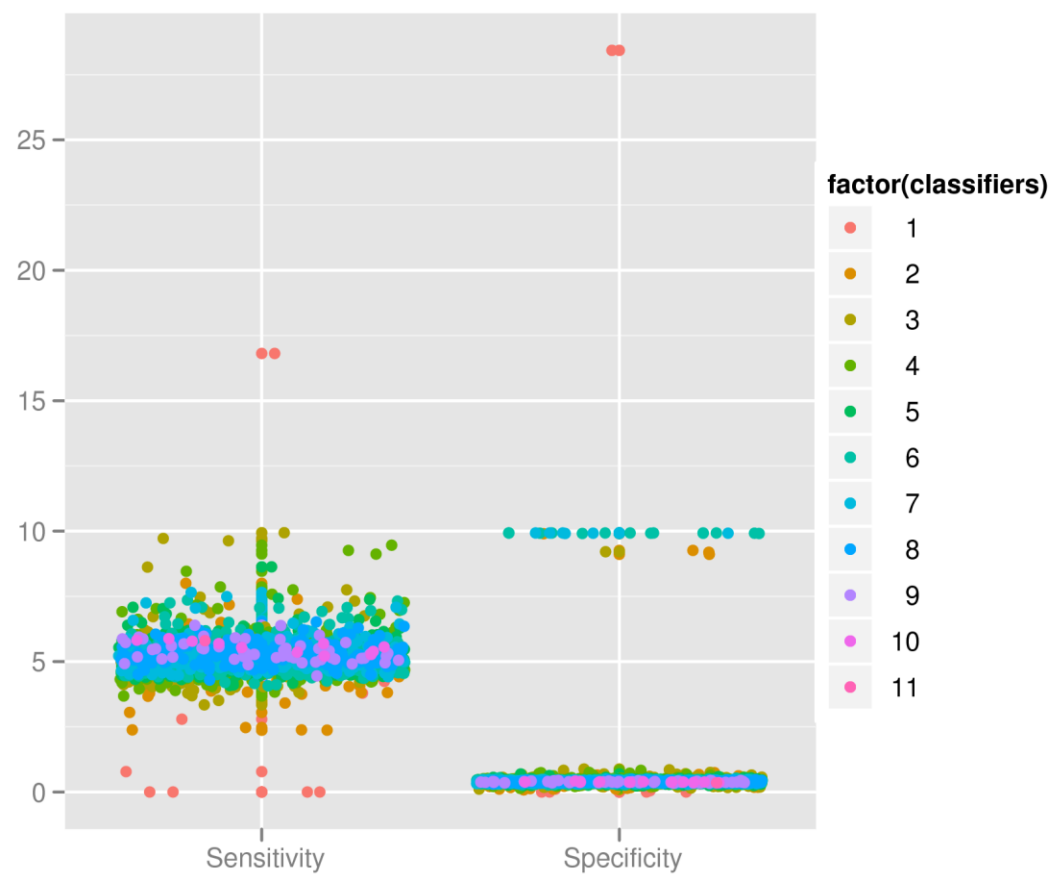

Figure S3

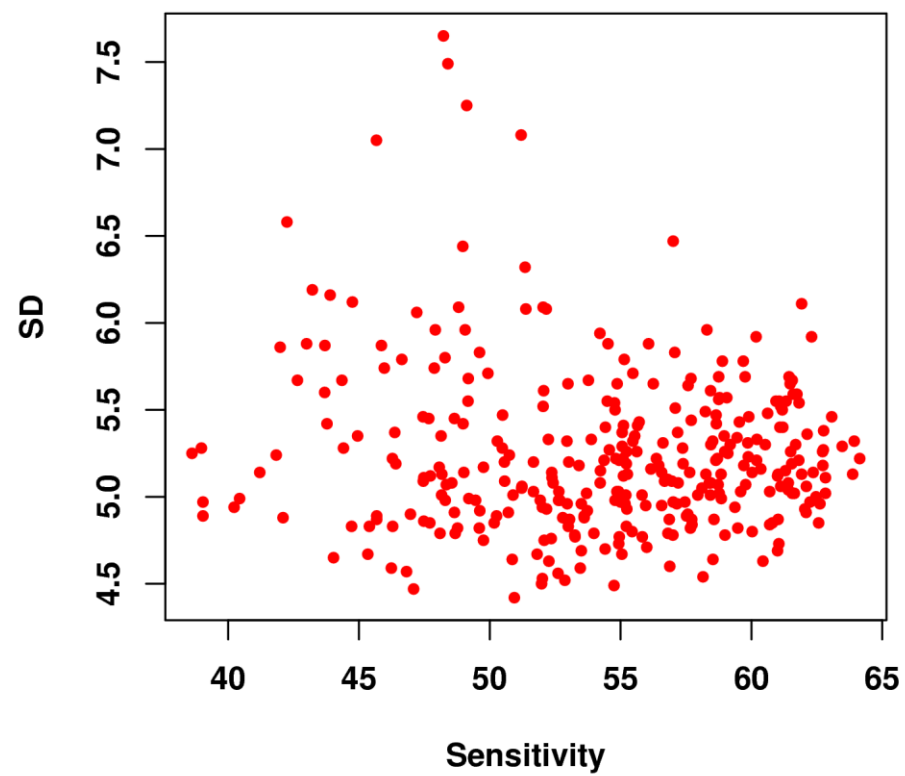

Supplement: Supplementary file 1 — Fig. S1. Taverna workflow (Hull et al. 2006; Oinn et al. 2006b) used to test the different combinations of prediction tools on the training and test sets of the known mitochondrial proteins. Fig. S2. Variability in the accuracy of predictions. Standard deviation (SD, from 100 training and test runs of the support vector machine) of the sensitivity (left) and specificity (right) for the 2047 possible combinations of prediction tools. The number of prediction tools used to determine each mean prediction is color coded. Fig. S3. Relationship between the mean sensitivity and variability (shown as SD, calculated from 100 independent training and test runs of the SVM) for different combinations of seven prediction tools. [file mmc1.pdf]
